# Fabrication of ɛ-Polylysine-Loaded Electrospun Nanofiber Mats from Persian Gum–Poly (Ethylene Oxide) and Evaluation of Their Physicochemical and Antimicrobial Properties

**DOI:** 10.3390/foods12132588

**Published:** 2023-07-03

**Authors:** Zahra Souri, Sara Hedayati, Mehrdad Niakousari, Seyed Mohammad Mazloomi

**Affiliations:** 1 Department of Food Hygiene and Quality Control, School of Nutrition and Food Sciences, Shiraz University of Medical Sciences, Shiraz 7193635899, Iran; zahra.souri@yahoo.com (Z.S.); mazloomi@sums.ac.ir (S.M.M.); 2 Nutrition Research Center, School of Nutrition and Food Sciences, Shiraz University of Medical Sciences, Shiraz 7193635899, Iran; 3 Department of Food Science and Technology, School of Agriculture, Shiraz University, Shiraz 7144165186, Iran; niakosar@shirazu.ac.ir

**Keywords:** Persian gum, Poly (ethylene oxide), ɛ-polylysine, electrospinning, nanofiber, antimicrobial

## Abstract

In the present study, electrospun nanofiber mats were fabricated by mixing different ratios (96:4, 95:5, 94:6, 93:7, and 92:8) of Persian gum (PG) and poly (ethylene oxide) (PEO). The SEM micrographs revealed that the nanofibers obtained from 93% PG and 7% PEO were bead-free and uniform. Therefore, it was selected as the optimized ratio of PG:PEO for the development of antimicrobial nanofibers loaded with ɛ-Polylysine (ɛ-PL). All of the spinning solutions showed pseudoplastic behavior and the viscosity decreased by increasing the shear rate. Additionally, the apparent viscosity, G′, and G″ of the spinning solutions increased as a function of PEO concentration, and the incorporation of ɛ-PL did not affect these parameters. The electrical conductivity of the solutions decreased when increasing the PEO ratio and with the incorporation of ɛ-PL. The X-ray diffraction (XRD) and Fourier-transform infrared (FTIR) spectra showed the compatibility of polymers. The antimicrobial activity of nanofibers against *Escherichia coli* (*E. coli*) and *Staphylococcus aureus* (*S. aureus*) was investigated, and the samples loaded with ɛ-PL demonstrated stronger antimicrobial activity against *S. aureus.*

## 1. Introduction

The microbial contamination of food products may cause spoilage and foodborne diseases. These pathogens have posed concerns in the food industry and to public health. Changing the sensory properties of food products and increasing the risk of hospitalization and death are the major adverse effects of food pathogens [1,2]. Foodborne microorganisms are affected by several factors such as moisture in food, acidity, amount of nutrients, temperature, humidity, oxygen, and light. Packaging protects food products against microbial contaminations and physicochemical damage during transportation and storage. Additionally, releasing active packaging with antimicrobial properties is considered to be an effective approach to increase the shelf life and safety of food products [3]. Therefore, the incorporation of antimicrobials into food packaging has increased in recent years. Scientists suggest the application of natural substances instead of synthetic materials to eliminate their unwanted adverse effects [4,5]. The application of natural antimicrobials in food packaging as a substitute for chemical preservatives improves the safety of food [6]. ɛ-PL is a natural and safe antimicrobial peptide compound with no side effects on humans. It is produced by *Streptococcus albulus*, which is decomposed to lysine, and can be considered a source of lysine amino acid [7]. ɛ-PL has gained considerable attention due to its broad antimicrobial spectrum against bacteria, fungi, and yeasts. Additionally, it is non-toxic, non-mutagenic, biodegradable, and does not have negative effects on the organoleptic properties of food products [8]. Other important features of this antimicrobial agent are its high stability in alkaline and acidic environments, high solubility in water, and heat resistance [9]. On the other hand, the non-biodegradability of petroleum-based packaging materials has caused environmental concerns and increased the demand for biopolymer-based food packaging materials. The edible films can be produced via different methods, such as casting, extrusion, and electrospinning. The latter is an electrohydrodynamic technique that uses a high electric field to fabricate micro- to nano-scale fiber mats [10]. Electrospinning is a cost-effective and easy technique that does not require thermal processing. Therefore, it can stabilize the bioactive agents in the polymeric substrate [11]. Moreover, electrospun structures have high porosity and a large surface area. These properties are desirable for the controlled release of bioactive compounds from the packaging matrix onto the surface of food [12]. Therefore, electrospinning is an appropriate method for fabricating biopolymer-based antimicrobial nanofibers. Persian gum (PG) is a biodegradable and inexpensive polysaccharide exudate from wild almond (*Amygdalus scoparia Spach*) shrubs which are grown in the central regions of Iran [13]. It is an acidic anionic hydrocolloid comprising a water-soluble and an insoluble fraction. This biopolymer has been applied in the development of edible films by several researchers [14,15]. However, it is not spinnable due to the high negative charge and the repulsion between biopolymer chains that prevents the formation of entanglements [16]. A combination of non-spinnable biopolymers with a spinnable polymer is a promising method for the development of electrospun nanofibers. A study by Keramat et al. [16] revealed that the incorporation of high levels of polyvinyl alcohol into Persian gum makes it spinnable. However, the obtained fiber mat was not uniform and had beads even in the presence of 70% polyvinyl alcohol. Poly (ethylene oxide) (PEO) is a synthetic, FDA-approved polymer for food and clinical applications. It is widely used for the production of electrospun nanofibers due to its good spinnability, mechanical stability, biocompatibility, biodegradability, non-toxicity, and high level of safety [17]. This polymer has been added to biopolymers such as peach gum [11], cellulose acetate [17], chitosan [18], and whey protein isolate [19] to make them spinnable. 

However, to the best of our knowledge there is not a published paper about the development of PG/PEO fiber mats. Therefore, in this study, antimicrobial electrospun fiber mats were fabricated using different ratios of PG and PEO (96:4, 95:5, 94:6, 93:7, and 92:8). Subsequently, ε-PL was added to the optimal ratio and the physicochemical and antimicrobial properties of fiber mats against *S. aureus* and *E. coli*, two of the most important and widely spread food pathogens, were investigated.

## 2. Materials and Methods

### 2.1. Materials

Persian gum was collected from wild almond shrubs in Shiraz, Iran. It contained 7.92% moisture, 2.21% ash, 0.18% fat, 0.22% protein, 87.88% total sugar, and 9.68% uronic acid. The average molecular weight of Persian gum was 4.15 × 10^6^ Da. Poly (ethylene oxide) (average molecular weight = 9 × 10^5^ Da) and ε-Polylysine were acquired from Sigma-Aldrich (St. Louis, MO, USA). The nutrient agar culture was obtained from Merck Chemicals Co. (Darmstadt, Germany). *E. coli* and *S. aureus* were procured from the Persian Type Culture Collection (PTCC).

### 2.2. Preparation of Spinning Solutions

A solution of PG in distilled water (5% *w*/*v*) was prepared and placed on a magnetic stirrer at room temperature for 24 h to be completely hydrated. PEO was added to distilled water to prepare a 5% *w*/*v* solution and magnetically stirred at 70 °C for 30 min. Then, different ratios of PG:PEO (96:4, 95:5, 94:6, 93:7, and 92:8) were mixed for 30 min with a magnetic stirrer at room temperature to obtain the spinning solutions. Finally, ε-PL(1 mg/mL) was added to the electrospinning solution of the sample, which produced the optimal nanofibers, and it was stirred for 5 min.

### 2.3. Characterization of the Electrospinning Solution

#### 2.3.1. Rheological Measurements

The rheological properties of spinning solutions were measured with a rheometer (MCR-302, Anton Paar, Graz, Austria). Steady shear data of spinning solutions were obtained at 25 °C, and shear rates were from 14 to 300 S^−1^. The dynamic oscillatory measurements were performed with a cone and plate geometry (1 mm gap and 25 mm cone diameter). The spinning solutions were poured into the rheometer cup and covered with silicon oil to prevent the evaporation of water during the experiments. Afterward, frequency sweeps tests were carried out within the frequency range of 0.1 to 100 Hz at 0.5% strain and 25 °C.

#### 2.3.2. Electrical Conductivity

The electrical conductivity (EC) of the spinning solutions was determined with a conductivity meter (CONSORT C933, Turnhout, Belgium) at 25 °C. Two electrodes with the same voltage were placed in the solution, and the current that it created, based on the conductive nature of the solution, showed the conductivity of the device.

### 2.4. Electrospinning Process

Nanofibers were fabricated with an electrospinning apparatus (NanoAzma, ES II, Tehran Iran) at 22 °C and 40% relative humidity. The instrument consisted of a high-voltage supply, a syringe pump, and a rotating collector. A 3 mL syringe with an 18-gauge needle was filled with the electrospinning solution and placed in front of the flow pump. The drum collector was covered with aluminum foil to collect the fiber mats. The spinning parameters were as follows: spinning voltage of 15 kV, injection rate of 1.3 mL/h, and receiving distance of 10 cm.

### 2.5. Characterization of Nanofibers

#### 2.5.1. Scanning Electron Microscopy (SEM)

To investigate the morphology of nanofibers with a scanning electron microscope, the resulting fibers were coated with gold using a sputter coater (DSR1, Nanostructural Coating Co., Tehran, Iran) and photographed with an SEM device (TESCAN Vega3, Brno, Czech Republic) at a voltage of 20 kV and a magnification of 3000.

#### 2.5.2. Fourier Transforms Infrared (FT-IR)

To identify the functional groups, chemical bonds, and molecular interactions, the FTIR spectra of nanofibers were obtained with an FTIR spectrometer (Tensor II, Bruker, Germany) with a wavelength resolution of 4 cm within the range of 4000 to 400 cm^−1^.

#### 2.5.3. X-ray Diffraction (XRD)

The crystal structure of nanofibers was investigated using an X-ray diffractogram (D8-Advance, Bruker, Karlsruhe, Germany) with a diffraction angle of 5 to 60 degrees, a voltage of 40 kV, and a current of 40 mA.

#### 2.5.4. Antimicrobial Properties of Nanofibers

The antibacterial properties of the PG/PEO nanofibers with and without ɛ-PL were investigated against two food pathogenic bacteria, *Staphylococcus aureus* (*S. aureus*) and *Escherichia coli* (*E. coli*). The bacterial inoculums (0.5 McFarland standard or 1.5 × 10^8^ CFU/mL) were cultured on nutrient agar plates. Round-shaped fiber mats (7 mm in diameter) were cut under sterile conditions and placed on inoculated plates. The plates were incubated for 24 h at 37 °C, and then the inhibition zone diameter around the nanofiber discs was determined with a caliper.

### 2.6. Statistical Analysis

The experiments were replicated three times, and the obtained data were reported as mean ± standard deviation. All statistical analyses were accomplished by the SPSS software (version 22, IBM-SPSS, New York, NY, USA). The data were submitted to an analysis of variance (ANOVA) and Duncan’s multiple range test, and *p* < 0.05 was considered significant.

## 3. Results and Discussion

### 3.1. Morphology of Nanofibers

**Figure 1:**
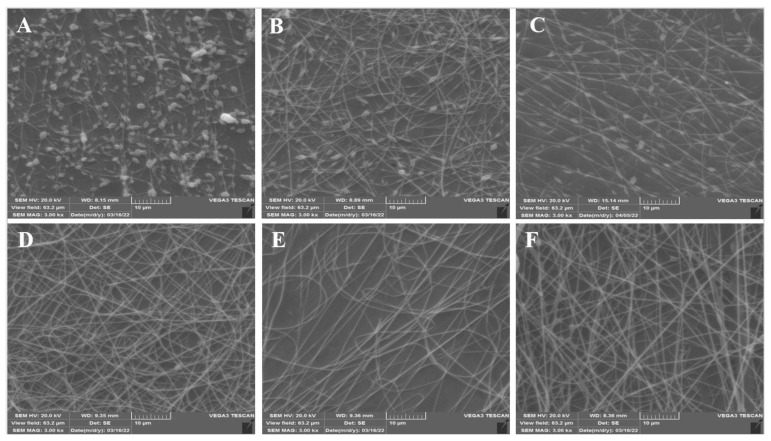
Scanning electron microscope images of PG/PEO nanofibers: (**A**) 96% PG and 4% PEO, (**B**) 95% PG and 5% PEO, (**C**) 94% PG and 6% PEO, (**D**) 93% PG and 7% PEO, (**E**) 93% PG, 7% PEO, with ɛ-PL, and (**F**) 92% PG and 8% PEO (scale bar = 10 µm).

The primary experiments revealed that pure PG solution is not spinnable. This behavior could be due to the strong repelling forces between biopolymer molecules that prevent the formation of sufficient chain entanglement. To increase the spinnability of PG, different ratios of PEO were blended with PG. PEO is a water-soluble, nontoxic, biodegradable, and biocompatible synthetic polymer that decreases the repulsive forces between molecules, modifies the electrical conductivity and viscosity of the spinning solution, and promotes the electrospinning process [20]. The lowest concentration of PEO required to obtain fibrous structures was 4%. However, the SEM images (Figure 1) revealed nonuniform and bead-like segments with very fine, non-integrated nanofibers. The electrospinning of biopolymers is a complicated procedure due to their composition, viscosity, and high molecular weight. Several factors, such as polymer blend ratio and concentration, viscosity, solubility, and electrical conductivity, affect the morphology of nanofibers. The morphology of electrospun samples changed from highly beaded structures to uniform structures as the ratio of PEO increased. This behavior was due to the high solubility of PG in PEO and the need to maintain the surface tension and viscosity of spinning solutions. PEO is a flexible chain polymer that interacts with biopolymers through hydrogen bonding. Also, the oxygen atoms of the PEO backbone contribute to the formation of sufficient chain entanglements that bind with the intermolecular interactions of biopolymers and facilitate their spinnability [21]. At low ratios of PEO concentrations, the polymeric solutions could not be electrospun due to weak hydrogen bonding and deficient chain entanglements between PG polymer chains and PEO. At higher ratios, the concentration of PEO was sufficient to interact with PG and form hydrogen bonds, disrupting the intermolecular interactions of PG. Therefore, smooth, bead-free, and uniform-diameter nanofibers were produced by electrospinning [21]. With appropriate polymer blends, the counterbalanced Coulombic stretching force led to the formation of beadless and continuous nanofibers [22]. The PG:PEO ratio of 93:7 was observed to ascertain the optimum ratio for the formation of continuous, uniform, and defect-free nanofibers. At higher concentrations of PEO (92:8), the fibers were bead-free and uniform but the diameter of the nanofibers increased. These findings were in accordance with the results reported by Yildiz et al. [18], who stated that increasing the PEO concentration increased the diameter of nanofibers due to the uncharged nature of PEO and the reduction in electrical conductivity.

The solution comprising 93% PG and 7% PEO was selected as the optimal electrospinning solution to produce the ɛ-PL-loaded PG/PEO nanofibers. The scanning electron micrographs of PG/PEO nanofibers with ɛ-PL revealed that the addition of ɛ-PL did not affect the spinnability of the fibers.

### 3.2. Rheological Properties of Electrospinning Solutions

#### 3.2.1. Steady Shear Viscosity

**Figure 2:**
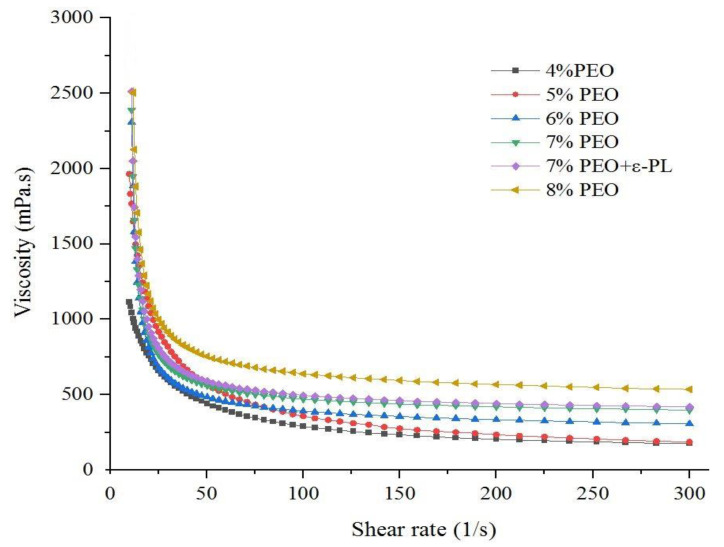
The viscosity of the PG/PEO spinning solutions is displayed according to their corresponding PEO contents.

The viscosity of the polymeric solution is an important factor in electrospinning because it has substantial effects on the formation of chain entanglement of the polymer molecules, which is required for the successful electrospinning of biopolymers [22]. As shown in Figure 2, all of the spinning solutions showed pseudoplastic behavior due to the shear-induced disruption of macromolecular chains and the alignment of randomly positioned intermolecular networks in the direction of shear [23]. The decrease in viscosity of the spinning solutions was more evident for the samples with higher levels of PG, indicating its high shear dependency. The pronounced viscosity reduction in PG could be due to the quicker alignment of the linear PG molecules. This means that PG increased the flowability and shear-thinning behavior of the spinning solution. Increasing the PEO concentration increased the apparent viscosity of solutions, and the sample with 8% PEO was the most viscous spinning solution. This result is in agreement with the findings reported by Lu et al. [20], who observed an increase in viscosity by increasing the ratio of PEO to sodium alginate. The increase in viscosity might be due to the formation of intermolecular interactions between PG and PEO. The samples with higher levels of PG solution were more sensitive to shear rate, and their viscosity reduction during shearing was more pronounced compared to the samples with higher levels of PEO. The addition of ε-PL to the solutions did not cause significant differences in the viscosity of spinning solutions. The effect of ε-PL on the viscosity of biopolymers was highly dependent on the electrical charge of hydrocolloids. The inclusion of ε-PL decreased the apparent viscosity of positively charged hydrocolloids such as chitosan [6,24,25]. This behavior was due to the increased repulsive forces between the molecules of biopolymers. While negatively charged hydrocolloids such as carrageenan have shown an opposite trend [26], this could be due to the electrostatic interactions between positively charged ε-PL and negatively charged biopolymers. However, we found no statistically significant differences in the rheological properties of the samples with and without ε-PL. This behavior could be explained the low concentration of ε-PL in our study.

#### 3.2.2. Viscoelastic Properties

**Figure 3:**
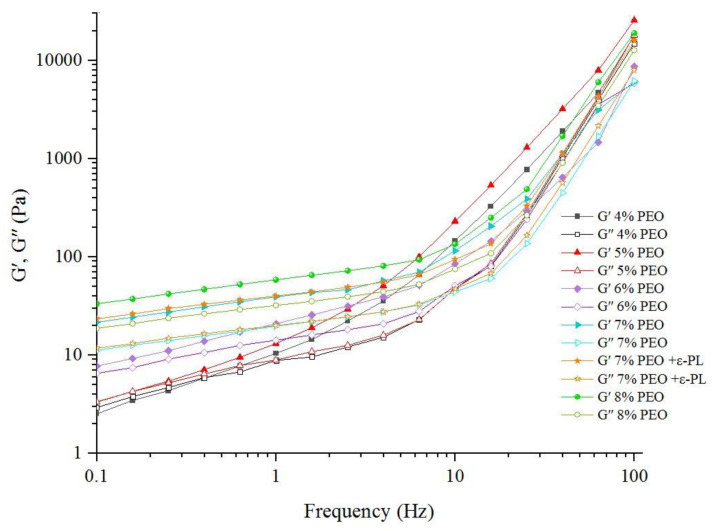
G′ and G″ values of the PG/PEO spinning solutions are displayed by their corresponding PEO contents.

The elastic (G′) and viscous (G″) moduli of the spinning solutions are presented in Figure 3. The samples with a low concentration of PEO had lower G′ and G″ values, and the differences between the G′ modulus and the corresponding G″ modulus were very small. Crossover occurred at low frequencies in the samples, with a low PEO ratio indicating its high-frequency dependency. The G′ and G″ values increased with the PEO concentration, and the G′ modulus was higher than the G″ modulus. Additionally, crossover occurred at higher frequencies in the samples with higher ratios of PEO, meaning that the frequency dependency of G′ and G″ decreased when increasing the PEO ratio in spinning solutions. The spinning solutions represented a weak gel network, and the addition of ε-PL did not affect the G′ and G″ values. These findings were supported by steady shear measurements and might be attributed to the low concentration of ε-PL in spinning solutions. The sample with 8% PEO showed the highest G′ and G″ values, and the differences between these two moduli were higher than in other samples. This behavior was attributed to the higher viscosity and hydration capacity of PEO compared to PG and the contribution of PEO to the development of an entangled network.

### 3.3. Electrical Conductivity of Electrospinning Solutions

**Figure 4:**
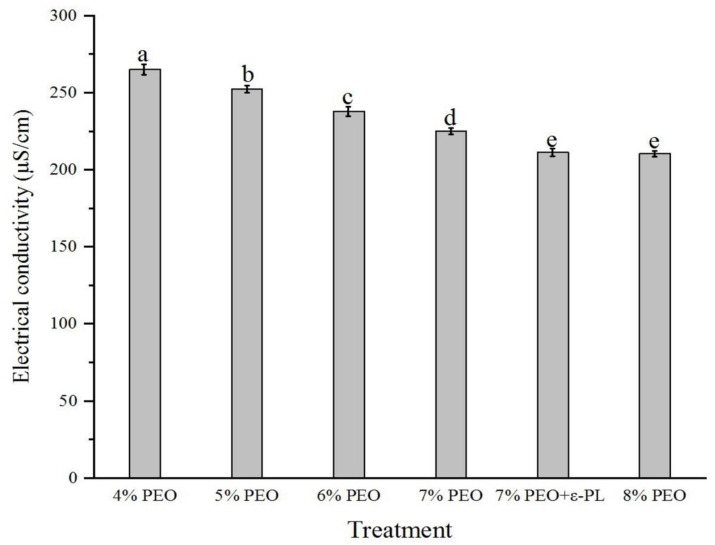
The electrical conductivity (µS/cm) of the electrospun PG/PEO nanofibers is displayed by their corresponding PEO contents. Different letters show significant differences between treatments.

The electrical conductivity of electrospinning solutions is a key factor in the fabrication of lead-free and uniform fibers. Sufficient electrical conductivity can overcome the surface tension of electrospun droplets, increase the electrostatic repulsion, facilitate the jetting process, and produce uniform fibers in electrospinning [27], while high electrical conductivity in biopolymers prevents their spinnability due to excessive stretching, which leads to an unstable polymer jet and the discontinuity of the electrospinning process [28]. The electrical conductivity of different electrospinning solutions is presented in Figure 4. The solution with the lowest concentration of PEO showed the highest conductivity, and a decreasing trend was observed in the electrical conductivity of spinning solutions upon increasing the PEO ratio. Yildiz et al. [18] reported that PEO is an uncharged polymer that reduces spinning solutions’ electrical conductivity. Biopolymers such as PG are considered polyelectrolytes, and their ion formation capability determines their electrical conductivity. The sample with the highest concentration of PG (PG:PEO 96: 4) formed fine, beaded fibers. Increasing the concentration of PEO increased the fiber diameter and decreased the beads. The increased viscosity of spinning solutions caused by increasing the concentration of PEO might have led to the fabrication of fewer beaded fibers. The addition of ε-PL to the spinning solutions decreased its electrical conductivity. The electrical conductivity of spinning solutions concerns the type of polymers and solvent and the presence of ions. The decreased electrical conductivity of solutions caused by the addition of ε-PL was because it is a positively charged polypeptide, while PG and PEO are negatively charged polymers. As a result, the addition of ε-PL reduced the electrical conductivity of solutions. These findings contradict the findings of Liu et al. [6], who claimed that ε-PL increased the conductivity of spinning solutions. This can be attributed to the differences in the types of spinning polymers. We used PEO and PG, which have a negative electrical charge, while they used chitosan, which is a positively charged polymer.

### 3.4. FTIR

**Figure 5:**
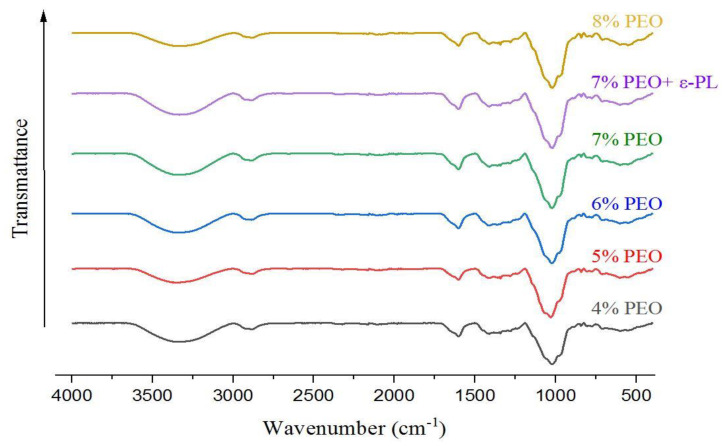
The FTIR spectra of the electrospun PG/PEO nanofibers are displayed by their corresponding PEO contents.

FTIR analysis was performed to study the compatibility of spinning polymers with each other. The FTIR spectra of nanofibers are presented in Figure 5. The stretching vibration band centered at 3323 cm^−1^ corresponds to the hydroxyl groups. The peak at 2927 cm^−1^ is related to the asymmetric stretching of CH_3_, while the band at 2859 cm^−1^ corresponds to the symmetric CH_2_ groups. The strong band at 1608 cm^−1^ is the indicator of COOH groups in uronic acid residues [29]. The absorbance band at 1417 cm^−1^ is related to the bending vibration of C–H. The peaks between 1200 and 1000 cm^−1^ are assigned to the C–O and C–C complex bands. The absorbance band at 1017 cm^−1^ is attributed to C–O or C–C functional groups, and the peak between 1000 and 700 denotes C–H vibrational bonds. The characteristic peaks of PG were observed in the FTIR spectra of nanofibers, and the peak at 1017 was intensified by the increase in the PEO ratio.

### 3.5. X-ray Diffraction (XRD) of Nanofibers

**Figure 6:**
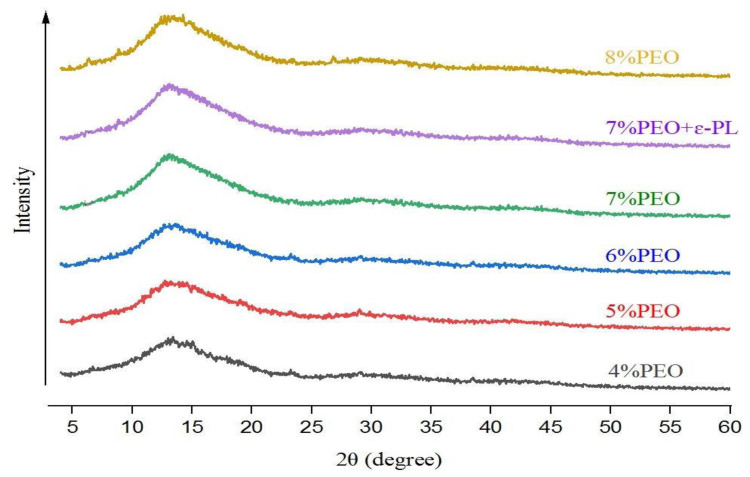
The X-ray diffraction patterns of electrospun PG/PEO nanofibers are displayed by their corresponding PEO contents.

The crystallographic structures of PG/PEO nanofibers with and without ɛ-PL are presented in Figure 6. Crystallinity is an indication of the mechanical strength of nanofibers, and generally the robustness of crystalline materials is higher than that of amorphous ones [30]. The X-ray diffractograms exhibited similar patterns for all of the nanofibers. The XRD patterns of PG/PEO exhibited a broad diffraction peak centered at 2θ = 13. The peak was intensified by the increase in the PEO ratio, and the highest peak was observed in the sample with 8% PEO. PG is an amorphous biopolymer and reduces the crystallinity of nanofibers. The typical sharp peaks of PEO at 19 and 23 diffraction angles of 2θ were not observed in XRD patterns due to the interference of PG with the molecular chain arrangement in PEO. These results agree with the results reported by Allafchian et al. [30], who demonstrated that PG reduces the crystallinity of PVA nanofibers by increasing the mobility of PVA polymeric chains and interfering with the development of the semi-crystalline structure. The addition of ɛ-PL to nanofibers did not change the diffraction pattern or peak intensity of the nanofibers. Although ɛ-PL is amorphous, its concentration in spinning solutions was very low and had no effect on the crystalline pattern of nanofibers.

### 3.6. Antimicrobial Activity

**Figure 7:**
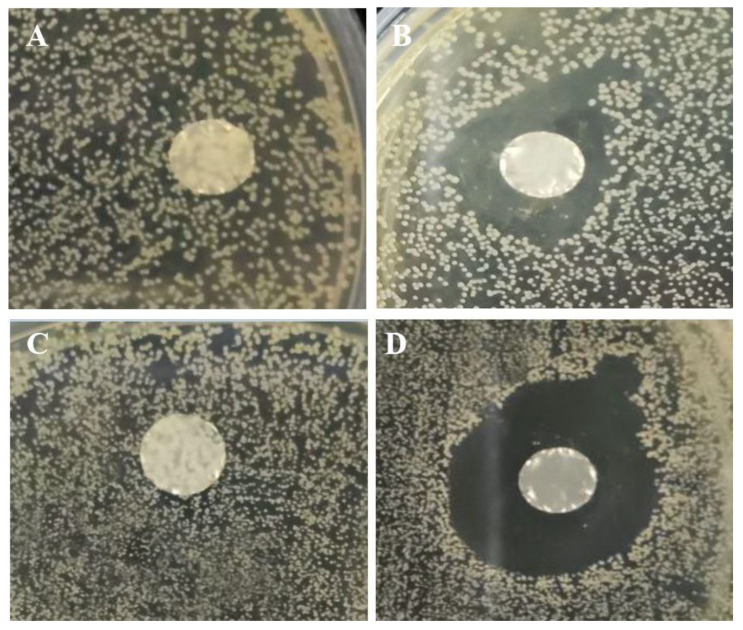
The antimicrobial effects of nanofibers: (**A**) PG/PEO against *E. coli*, (**B**) PG/PEO/ɛ-PL against *E. coli*, (**C**) PG/PEO against *S. aureus*, and (**D**) PG/PEO/ɛ-PL against *S. aureus*.

The antimicrobial activity of ɛ-PL-loaded fiber mats versus control nanofibers was investigated against two common foodborne pathogens, Gram-positive *S. aureus* and Gram-negative *E. coli*. Figure 7 displays significant visual differences in the bacterial cultures between the nanofibers with and without ɛ-PL. The control PG/PEO fiber mat did not show any inhibition zone against bacterial strains, while the sample with ɛ-PL showed an inhibition zone against *E. coli*. and *S. aureus*. This indicates the ability of ɛ-PL to inhibit bacterial growth. These findings are in line with the results reported by Lin et al. [31], who reported that ε-PL/chitosan nanofibers represented substantial antibacterial activity. The antimicrobial properties of ɛ-PL are attributed to the cationic nature of this polypeptide, which interacts with the negatively charged cytoplasmic membrane. These interactions destroy the cell wall structure, leading to cell death and inhibiting microbial contaminants [32]. Additionally, ɛ-PL affects the phospholipid bilayer of the cell membrane, decreases the polarity, and increases the cell membrane permeability, which leads to the release of cellular components such as proteins and ions. ɛ-PL interferes with the synthesis of proteins in bacterial cells or damages the bacterial cells via the destruction of cellular proteins and the aggregation of ɛ-PL by the bacterial proteins. According to Figure 7B,D the inhibition zone diameter against *E*. *coli*. was 13 ± 0.5 mm, while it was 17 ± 0.5 mm for *S. aureus*, indicating that the Gram-negative *E*. *coli*. was more resistant to ɛ-PL than the Gram-positive *S. aureus*. The Gram-negative bacteria have a lipopolysaccharide outer membrane, which may have a protective effect against antimicrobial agents. These findings support the findings of Li et al. [7], who stated that the differences in antimicrobial properties of ɛ-PL against Gram-negative and Gram-positive bacteria are due to differences in cell membrane structure and constituents.

## 4. Conclusions

In conclusion, the nanofiber mats were successfully produced from PG and PEO using the electrospinning technique. The experiments revealed that PEO is compatible with PG for electrospinning and can be considered a suitable polymer for making PG solution blends for electrospinning. A low concentration (7%) of PEO is enough to produce bead-free and uniform nanofibers. It was also found that antimicrobial fiber mats can be produced by the incorporation of ɛ-PL. These nanofibers could prevent the growth of *Staphylococcus aureus* and *Escherichia coli*, two important food pathogens. Therefore, PG/PEO/ɛ-PL nanofiber mats have good potential for active food packaging.

## Data Availability

The data presented in this study are available upon request from the corresponding author.
